# Stereotactic Body Radiation Therapy for Clinically Localized Prostate Cancer in Men With Hip Prostheses: A Cautionary Note

**DOI:** 10.7759/cureus.61432

**Published:** 2024-05-31

**Authors:** Sarthak Shah, Sanjeev Saravanakumar, Dylan Conroy, Srinivas Sowmiyanarayanan, Rahul Singh, Abigail Pepin, Harris Rashid, Malika T Danner, Pranay Krishnan, Siyuan Lei, Abdul Rashid, Simeng Suy, Shaan Kataria, Nima Aghdam, Sean Collins

**Affiliations:** 1 Radiation Oncology, Rutgers Cancer Institute of New Jersey, New Brunswick, USA; 2 Radiation Oncology, MedStar Georgetown University Hospital, Washington, USA; 3 College of Arts and Sciences, Emory University, Atlanta, USA; 4 College of Arts and Sciences, Case Western Reserve University, Cleveland, USA; 5 Radiation Oncology, University of Pennsylvania Abramson Cancer Center, Philadelphia, USA; 6 Radiology, MedStar Georgetown University Hospital, Washington, USA; 7 Radiation Oncology, Arlington Radiation Oncology, Reston, USA; 8 Department of Radiation Oncology, Beth Israel Deaconess Medical Center, Harvard Medical School, Boston, USA

**Keywords:** toxicities, local recurrence, cyberknife, hip replacement, sbrt, prostate cancer

## Abstract

Purpose: Stereotactic body radiation therapy (SBRT) has been established as a safe and effective treatment for prostate cancer. SBRT requires high accuracy to reduce treatment margins. Metal hip prostheses create artifacts that distort pelvic imaging and potentially decrease the accuracy of target/organ at risk (OAR) identification and radiation dose calculations. Data on the safety and efficacy of SBRT after hip replacement is limited. This single-institution study sought to evaluate the safety and local control following SBRT for prostate cancer in men with hip replacements.

Methods: 23 patients treated with localized prostate cancer and a history of pre-treatment hip replacement, treated with SBRT from 2007 to 2017 at MedStar Georgetown University Hospital were included in this retrospective analysis. Treatment was administered with the CyberKnife^®^ (Accuray Incorporated, Sunnyvale, CA) at doses of 35 Gy or 36.25 Gy in 5 fractions. The targets and OARs were identified and contoured by a single experienced Radiation Oncologist (SPC). The adequacy of the CT and T2W MRI images for treatment planning was assessed with a three-point scale (good, adequate, or suboptimal). During treatment planning, care was taken to avoid treatment beams that directly traversed the hip prosthesis. Toxicities were recorded and scored using the Common Terminology Criteria for Adverse Events version 4.0 (CTCAE v.4.0). Local recurrence was confirmed by magnetic resonance imaging and/or prostate biopsy.

Results: The median follow-up was seven years. The patients were elderly (median age = 71 years) with a high rate of comorbidities (Charlson Comorbidity Index > 2 in 25%). Four patients had bilateral hip replacements. The majority of patients were low to intermediate risk per the D’Amico classification. Around 13% received upfront ADT. In total, 13 patients were treated with 35 Gy, and 10 were treated with 36.25 Gy. The rates of late > Grade 3 GU toxicity and > Grade 2 GI toxicity were 8.6% and 4.3%, respectively. There were no Grade 4 or 5 toxicities. Six patients (26%) developed a local recurrence at a median time of 7.5 years. Of these six patients, four had unilateral hip replacements and two had bilateral. Three underwent salvage cryotherapy and three received salvage ADT.

Conclusions: In the general population, high-grade toxicities and local recurrences are uncommon following prostate SBRT. However, in this cohort of patients with prior hip replacements, prostate SBRT had higher than expected rates of late toxicity and local recurrence. In the opinion of the authors, such patients should be counseled regarding an elevated risk of late toxicity and local recurrence with prostate SBRT. With its ultrasound guidance, brachytherapy would have the advantage of circumventing the need for MRI/CT-based imaging and thus may represent a preferable radiation alternative in this patient population. If these patients are treated with SBRT, they should be monitored closely for local recurrence so early salvage can be performed. We hope that recent advances in metal artifact reduction techniques and dose-calculation algorithms will improve future outcomes.

## Introduction

Stereotactic body radiation therapy (SBRT) is known for its increased accuracy, intrafraction image guidance, and reduced treatment margins, enabling effective and convenient delivery of external radiation therapy [1]. SBRT enables clinicians to deliver high doses of radiation to the prostate while minimizing doses to adjacent normal structures in order to achieve higher tumor control with low morbidity [2]. Because SBRT uses small treatment margins and with steep dose gradients, it is important to verify that the prescribed dose is being delivered to the prostate [3, 4]. There are steep dose-response relations for toxicity [5] and local tumor control [6] of prostate cancer. Based on the steepness of the local control dose-response curve, an accuracy of 7% for dose delivery would be necessary to assure the predicted local control rate [7,8]. 

Symptomatic hip osteoarthritis is very common in elderly patients [9]. Standard management involves oral analgesics and physical therapy. Total hip replacement (THR) is the most common surgery performed for joint pain/stiffness that is refractory to conservative management. Several hundred thousand hip replacements are performed in the US each year for this condition and the number is increasing. The median age for hip replacement in the US is 69. THR use amongst elderly cancer patients is expected to increase in the future [10]. Hip prostheses are commonly made of high atomic number (Z) materials that may interfere with radiation therapy planning or delivery.

**Figure 1:**
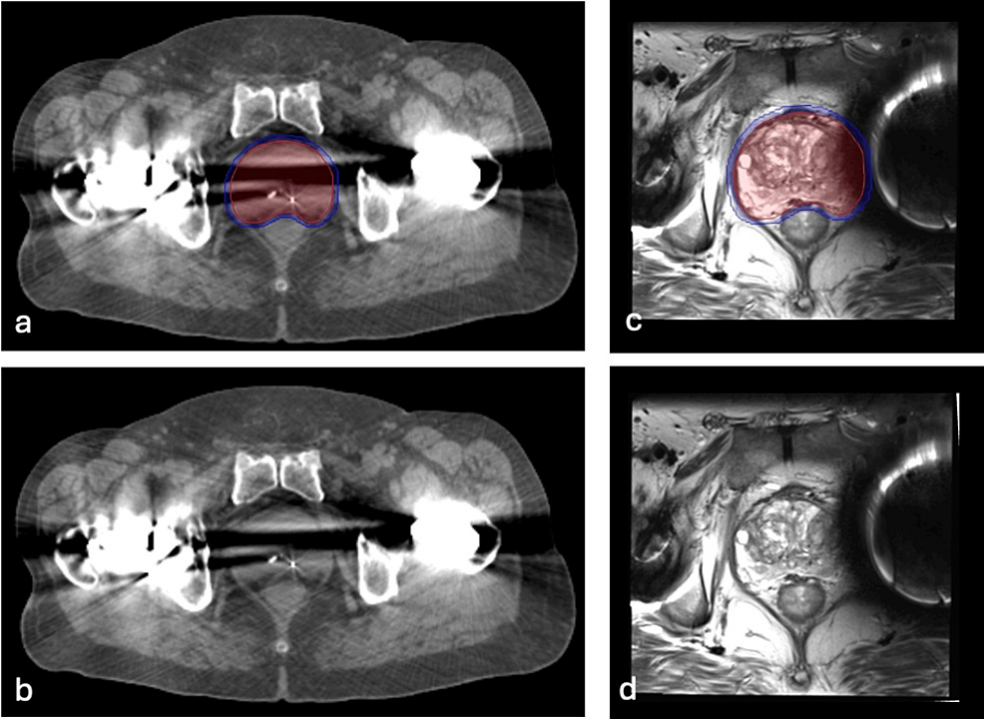
Treatment Planning Images Treatment planning sagittal computed tomography (CT) and magnetic resonance images (MRI) in a 71-year-old man with intermediate-risk prostate cancer and bilateral hip replacements treated with SBRT. The prostate (red) and 100% of the prescription dose (blue) are shown above. (a) CT with contours. (b) CT without contours. (c) MRI with contours. (d) MRI without contours.

At baseline, accurately contouring pelvic structures is challenging [11] with higher intra-observer variability than many other disease sites [12,13]. Hip replacements are an additional challenge because they create CT and MRI imaging artifacts that can obscure pelvic anatomy and impair the ability of the treatment planning system to determine the electron density for dose modeling [14]. CT imaging artifacts including streaking and blurring are even more severe in patients with bilateral hip replacements (Figure 1) [15]. Treatment planning MRIs are commonly employed which may improve soft tissue delineation in patients with hip prosthesis [15,16]. Treatment planning T2W MRI scans allows for smaller treatment volumes [17]. However, they are also prone to distortions in the presence of metallic prostheses. 

Patients with implanted hardware are commonly excluded from clinical trials. Thus, data regarding the safety and efficacy of SBRT after hip replacement is limited. The goal of our study is to report radiation-related toxicities and local control in patients with a history of hip replacement and SBRT to treat prostate cancer. This article was previously presented as a meeting poster at the 2022 ESTRO Annual Meeting on May 8th, 2022.

## Materials and methods

Patient selection

The Georgetown University Institutional Review Board (IRB) approved this single institution retrospective review of prospectively collected data (IRB#: 2009-510). All individuals diagnosed with localized prostate cancer who received SBRT at MedStar Georgetown University Hospital from 2007 to 2017 were eligible for inclusion. For inclusion in this study, patients were required to have received at least one hip implant with a minimum of 36 months follow-up post SBRT. Figure 1 demonstrates an axial section of treatment planning CT and MRI scans in a sample patient with bilateral hip replacements.

SBRT treatment planning and delivery

The institutional protocol was used for simulation, contouring, and treatment planning [18,19]. Treatment planning CT and T2W MRIs were obtained at least one week after insertion of 4 to 6 gold fiducial markers in the prostate. Treatment planning MRI was fused to the CT via fiducial alignment. The prostate and seminal vesicles comprised the clinical target volume (CTV). The planning total volume (PTV) was found by increasing the CTV by 3 mm in the posterior direction and 5 mm in the other directions. The prescription dose of 35-36.25 Gy was delivered to the PTV over one to two weeks in five fractions of 7-7.25 Gy each. Dose inhomogeneity corrections were not utilized. When available, Monte Carlo dose calculations were employed for plan verification [20]. Multiplan (Accuray Inc., Sunnyvale, CA) inverse treatment planning with dose-volume histogram analysis was utilized to evaluate the bladder and membranous urethra after contouring and treatment planning. The empty bladder volume receiving 37 Gy was limited to < 5 cc. The membranous urethra dose-volume histogram (DVH) goal was < 50% of the volume receiving 50% of the prescribed dose. The prescription isodose line was limited to > 75% which limited the maximum prostatic urethra dose to 133% of the prescription dose [18]. Target position was verified multiple times during each treatment using paired, orthogonal X-ray images which avoid hip prostheses. When possible, the artificial hip’s composition was obtained.

Follow-up and statistical analysis

**Figure 2:**
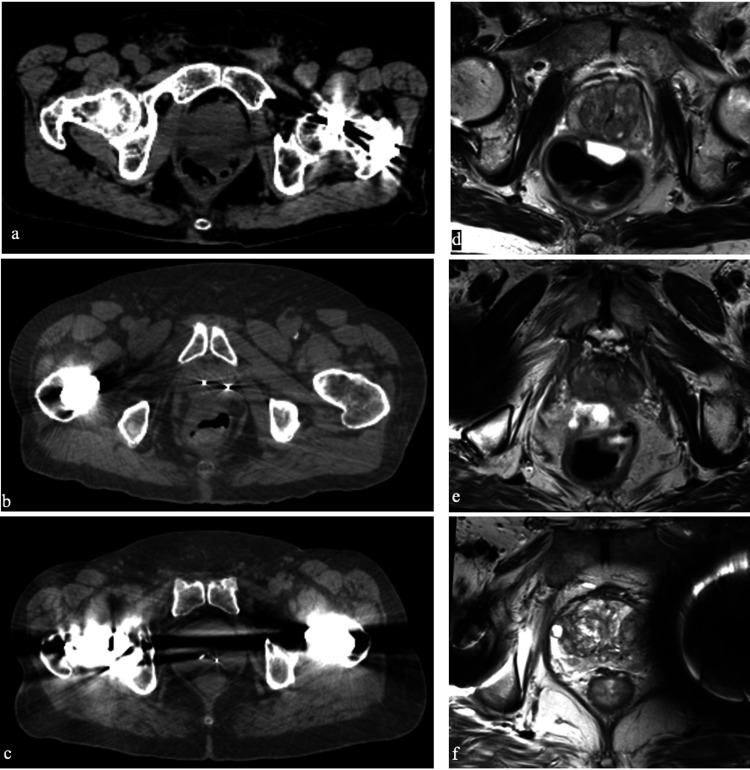
Representative CT and MR treatment planning images, demonstrating: (a) Good-quality CT with unintrusive streak artifact. (b) Adequate-quality CT with unintrusive streak artifact. (c) Suboptimal-quality CT with intrusive streak and banding artifact. (d) High-quality MRI without distortion. (e) Adequate-quality MRI with peri-prosthesis distortion. (f) Suboptimal-quality MRI with intrusive distortion.

The adequacy of the CT and T2W MRI images for treatment planning was blindly retrospectively evaluated by a radiation oncologist (SPC) with 15 years of experience using a three-point scale. A score of 0 was defined as good; a score of 1 was defined as adequate and a score of 2 was defined as suboptimal (Figure 2). Toxicities were assessed and PSA levels were obtained prior to treatment, every three months post-SBRT for one year, every six months for the following two years, and then yearly. Toxicities were assessed using the common terminology for adverse events version 4 (CTCAE v4). Grade 2 toxicity is generally designated as symptoms that require medications such as alpha-antagonists and antidiarrheal medications, or laser coagulation. A Grade 3 toxicity indicates minor surgical intervention (i.e., urethral dilation) was required for complications. The Kaplan-Meier method was utilized to determine actuarial likelihood estimates for toxicities. If PSA levels rose to >1 ng/ml, a digital rectal exam (DRE) was performed and a mpMRI was then obtained [21]. If abnormalities were noted in either the DRE or mpMRI, a biopsy was recommended.

## Results

From February 2009 to January 2014, 23 patients with pre-SBRT hip replacements were treated with SBRT for their prostate cancer (Table 1). The median follow-up was seven years. Patients were ethnically diverse, with 35% of Black ancestry and 4% of Hispanic ancestry. The median age was 71.5 years, with a range of 62 to 85 years. Four patients had bilateral hip replacements. Comorbidities were common (Charlson Comorbidity Index (CCI) > 2 in 25%). 13% were on anticoagulants and 17.4% had prior procedures for benign prostatic hyperplasia (BPH). Six patients had low-risk disease, 15 patients had intermediate-risk disease, and two patients had high-risk disease by D’Amico classification. Three patients (13.0%) received androgen deprivation therapy (ADT). Around 57% of the cohort were treated with 35 Gy in five fractions. The remainder was treated with 36.25 Gy in five fractions [22]. Six patients (25%) developed a local recurrence at a median time of 7.5 years.

**Table 1:** Patient and treatment characteristics PSA: Prostate-specific antigen

Characteristics	Patient values (n = 23)
Median Age (years)	71
Race	
White	61.0%
Black	35.0%
Hispanic	4.0%
D’Amico Risk Group	
Low	26.0%
Intermediate	65.0%
High	8.7%
Clinical Stage	
T1c – T2a	82.6%
T2b+	17.4%
Gleason Score	
5	4.3%
6	30.4%
7	56.5%
8	8.8%
Median PSA (ng/mL) at diagnosis	6.5
ADT	
No ADT	87.0%
ADT	13.0%
Treatment Dose	
35 Gy	57.0%
36.25 Gy	43.0%
Hip Prosthesis	
Unilateral	82.6%
Bilateral	17.4%
Treatment Planning MRI	
Yes	82.6%
No	17.4%

Evaluation of the CT scans and T2W MRIs is shown in Tables 2-3. Scoring of scans revealed 87% of CT scans adequate or above, with all 23 CT scans evaluated to have streaking present, and 43% of CT scans having banding. All T2W MRIs were shown to be adequate or above. However, 32% of MRIs had distortion.

**Table 2:** Quality of images from imaging questionnaires

Image Quality	Good	Adequate	Suboptimal
CT	13% (3)	74% (17)	13% (3)
MRI	63% (12)	37% (7)	0% (0)

**Table 3:** Image artifacts from imaging questionnaires

Image Artifacts	Yes	No
CT streaking	100% (23)	0% (0)
CT banding	43% (10)	57% (13)
MRI distortion	32% (6)	68% (13)

Actuarial incidence rates of acute and late GU and GI toxicities are demonstrated in Table 4. The incidence rates of > Grade 2 acute GU toxicity and > Grade 2 acute GI toxicity were 35% and 26% respectively. There were no acute Grade 3, 4 or 5 toxicities. The incidence rates of > Grade 3 late GU toxicity and > Grade 2 late GI toxicity were 8.6% and 4.3%, respectively. There were no late Grade 4 or 5 toxicities.

**Table 4:** Acute and late GI and GU toxicities GI: gastrointestinal, GU: genitourinary

Toxicity	None	Grade 1	Grade 2	Grade 3
Overall Acute				
GU	52% (12)	13% (3)	35% (8)	0% (0)
GI	43% (10)	30% (7)	26% (6)	0% (0)
Overall Late				
GU	22% (5)	13% (3)	57% (13)	8.6% (2)
GI	74% (17)	22% (5)	4.3% (1)	0% (0)

## Discussion

Successful prostate cancer treatment depends on the prescribed dose being delivered accurately. Small decrements can lead to high rates of toxicity and low rates of local control [6,7]. To date, there is limited data on toxicity and local control following radiation therapy in patients with hip replacements. In this study, the rates of Grade 2 and higher toxicities were higher than that previously published for prostate SBRT [2, 18, 22]. The cohort in this study was elderly with comorbidity being common prior to treatment. Specifically, 25% of patients had greater than two comorbid conditions, 13% were on anticoagulants and 17% had prior TURP. It has been reported that patients with high comorbidity scores are at increased risk of radiation therapy-related toxicity [23]. Another likely reason for toxicity is the higher uncertainty in the location of the bladder neck and anterior rectal wall in relation to the prostate, as hip replacements distort imaging. As expected, this is likely related to poor visualization of soft tissue structures. There were two patients with bulbar strictures that were treated with dilation. Three patients experienced late rectal bleeding and were treated with coagulation. Both of these complications did not recur. All three patients with bleeding were taking anticoagulants at the time of rectal bleeding [24].

The mechanism of these increased local failures is likely multifactorial including treatment planning errors and tight margins [25]. Poor local control could be due to decreased PTV dose from inaccurate dose calculations. Contouring inaccuracy is a major source of error in RT delivery. High Z metal hip prosthesis causes streaking and blurring CT artifacts that may cause difficulties in delineating the target volume/critical structures and may reduce dose calculation accuracy [26]. Metal artifact reduction methods and dose calculation algorithms are available but may not be adequate [27]. 

There is no consensus on how to irradiate patients with hip prostheses and if patients with hip implants should be excluded from SBRT, and the optimal SBRT dose is unknown. A common approach is to avoid beams traversing the prosthesis, and it is critical to use treatment fields that avoid the prosthesis. Treatment planning software is utilized to minimize artifacts from a metal prosthesis on CT images. Utilization of Monte Carlo dose calculations for the prosthesis and surrounding structures is critical when checking treatment plans. [28]. Providers planning treatments for patients with hip prostheses can consider an in vivo dosimeter to measure the daily dose delivered [29]. Due to the concern for increased toxicity and unknown benefit of dose escalation, half the patients in this series were treated with 35 Gy [30]. Recent data suggest that 40 Gy is ideal [31]. Local recurrences are difficult to identify and commonly occur many years (> 10 years) after the completion of treatment [6].

Limitations

This study had various limitations. The number of patients with hip replacements was small as brachytherapy is the preferred treatment for these patients at our institution. Additionally, Monte Carlo calculations were only utilized in patients at the discretion of the treating physician, which led to a lack of uniformity in the protocol. Our results may not be applicable to the general patient population due to the level of comorbidities in these patients, which may have contributed to toxicities. Finally, these patients were treated over a decade, and not all treatment plans utilized metal artifact reduction techniques and/or modern dose-calculation algorithms. 

## Conclusions

In the general population, high-grade toxicities and local recurrences are uncommon following prostate SBRT. However, in this cohort of patients with prior hip replacements, prostate SBRT had higher than expected rates of late toxicity and local recurrence. In the opinion of the authors, such patients should be counseled regarding an elevated risk of late toxicity and local recurrence with prostate SBRT. With its ultrasound guidance, brachytherapy would have the advantage of circumventing the need for MRI/CT-based imaging and thus may represent a preferable radiation alternative in this patient population. If these patients are treated with SBRT, they should be monitored closely for local recurrence so early salvage can be performed. It is hoped that recent advances in metal artifact reduction techniques and dose-calculation algorithms will improve future outcomes.
